# Determining structures of individual RNA conformers using atomic force microscopy images and deep neural networks

**DOI:** 10.21203/rs.3.rs-2798658/v1

**Published:** 2023-06-07

**Authors:** Maximilia F. S. Degenhardt, Hermann F. Degenhardt, Yuba R. Bhandari, Yun-Tzai Lee, Jienyu Ding, William F. Heinz, Jason R. Stagno, Charles D. Schwieters, Jinwei Zhang, Yun-Xing Wang

**Affiliations:** 1 Protein-Nucleic Acid Interaction Section, Center for Structural Biology, National Cancer Institute; Frederick, USA.; 2 Optical Microscopy and Analysis Laboratory, Cancer Research Technology Program, Frederick National Laboratory for Cancer Research, Frederick, MD 21702, USA; 3 Computational Biomolecular Magnetic Resonance Core, National Institute of Diabetes and Digestive and Kidney Diseases, National Institutes of Health; Bethesda, USA.; 4 Structural Biology of Noncoding RNAs and Ribonucleoproteins Section, Laboratory of Molecular Biology, National Institute of Diabetes and Digestive and Kidney Diseases, National Institutes of Health; Bethesda, USA.

## Abstract

The vast percentage of the human genome is transcribed into RNA, many of which contain various structural elements and are important for functions. RNA molecules are conformationally heterogeneous and functionally dyanmics^1^, even when they are structured and well-folded^2^, which limit the applicability of methods such as NMR, crystallography, or cryo-EM. Moreover, because of the lack of a large structure RNA database, and no clear correlation between sequence and structure, approaches like AlphaFold^3^ for protein structure prediction, do not apply to RNA. Therefore determining the structures of heterogeneous RNA is an unmet challenge. Here we report a novel method of determining RNA three-dimensional topological structures using deep neural networks and atomic force microscopy (AFM) images of individual RNA molecules in solution. Owing to the high signal-to-noise ratio of AFM, our method is ideal for capturing structures of individual conformationally heterogeneous RNA. We show that our method can determine 3D topological structures of any large folded RNA conformers, from ~ 200 to ~ 420 residues, the size range that most functional RNA structures or structural elements fall into. Thus our method addresses one of the major challenges in frontier RNA structural biology and may impact our fundamental understanding of RNA structure.

Knowledge about RNA structure and dynamics is important for understanding functions ^1,4,5^, designing novel devices ^6^ and developing RNA-targeting compounds ^7^. Since the first three-dimensional (3D) structures of tRNA were determined almost a half-century ago ^8,9^, there has been a slow increase in the number of RNA structures determined by NMR, X-ray crystallography, and most recently, cryo-EM ^10^, the backbone technologies for high-resolution structure determination. All these methods rely on signal-averaging and thus are limited to conformationally homogeneous samples. Since RNA molecules are dynamic and conformationally heterogeneous shown by direct visualization under physiological conditions ^2^, even for RNA as small as the HIV TAR RNA ^11^, a single snapshot structure falls short of accurately describing the conformational landscape ^12^. This limitation explains in part both the scarcity and redundancy of RNA structures in structural databases ^10,12^. Thus, given the rapid progress in RNA research and widespread applications in the biomedical and public health sectors, developing a method for studying the highly heterogeneous conformational space of RNA becomes ever-important and urgent.

AFM topographic images provide direct global structural information with a high signal-to-noise ratio (S/N) enabling visualization of individual molecules at a resolution sufficient to discern duplex helical grooves ^13–15^. The usefulness of global structural information in restraining RNA structures was previously implicated ^16,17^. However, the correlation between a topographic AFM image and the underlying atomistic topological structure, and its use for recapitulation of individual RNA conformer structures have not been explored. Importantly, AFM images of individual particles represent individual conformers of single molecules, and thus could in principle make it possible to study the conformations of individual molecules without signal-averaging. This utility is akin to other single-molecule methods such as the widely used single-molecule Förster resonance energy transfer (smFRET) ^18^, which has deconvoluted heterogeneous populations in numerous protein and RNA systems but is limited to providing sparse spatial information as only the conjugated fluorophores are visible.

In this report, we present HORNET (Holistic RNA structure determination method using AFM, unsupervised machine learning (ML), and deep neural Networks (DNN)), a novel approach for determining the individual three-dimensional (3D) topological structures of heterogeneous RNA conformers based on AFM data (Fig. 1). Our method drives the conformational trajectory toward a convergence that satisfies both the weighted AFM pseudo-potentials and classical Gibbs free-energy descriptions ^19,20^ (Fig. 1a; see Methods). The top structures are then selected and evaluated by both unsupervised and deep learning using a holistic consideration of all energetic and topographic information (Fig. 1b). Furthermore, we used deep neural networks (DNN) trained by a pseudo-structure database to provide an accuracy estimation of top structures in terms of root-mean-square-deviation (RMSD).

## Images of individual RNA conformers in solution

We use RNase P RNA from *Bacillus stearothermophilus* as an example to illustrate the utility of AFM (Fig. 2a–e, and Extended Data Fig. 3). These three topographic images (Fig. 2a) capture three individual RNA molecules (P1, P2, and P3) in three different conformational states, none of which is identical to the crystal structure (Fig. 2c). The background noise of the particle topography is between 1–5% of the maximum height (Extended Data Fig. 4).

The resolution of the P1, P2 and P3 images was assessed by applying the low-pass Fourier filter ^21^ (Extended Data Fig. 5) where clear spatial information at 0.87nm^−1^ (11.5 Å), 0.90nm^−1^ (11.1 Å), and 0.80nm^−1^ (12.5 Å) for P1, P2, and P3, respectively (Fig. 2d–e). The low resolution would seem to limit its use in structure determination. However, the RNA structural characteristics make AFM an ideal technique for the following reasons. RNA structure folding is hierarchical ^22^ and modular. Highly conserved A-form duplexes, which count for more than 70% of the mass in RNA structures in structural databases, vary within ~1.5 Å in terms of the backbone RMSD ^23^. Thus, in principle, given an initial structure as prior knowledge, 3D topological structures can be recapitulated from AFM molecular surfaces using a dynamic fitting with uncertainty significantly less than the inherent resolution limits of the AFM data itself, an approach similar to that used in X-ray crystallography and cryo-EM ^24^.

No structures of RNA have been determined using information solely from an individual macromolecule, as all reported structures were determined using signal-averaging methods. Thus, we first demonstrated our method by using a set of simulated data. Excluding structures of ribosomes and other protein-RNA complexes, there are only four classes of RNA larger than 210 nucleotides (nt) with resolutions better than 3.5 Å. They are the holo adenosylcobalamin riboswitch, Group I and Group II introns, and RNase P RNA. RNase P is a multi-turnover ribozyme that processes the 5’-ends of pre-tRNA and other RNAs ^25–27^. Its substrate promiscuity suggests conformational flexibility required for substrate binding and recognition ^25^. Studies using chemical probing and SAXS show that such large conformational changes, which can be as large as 30 Å ^25,28–30^, occur via the motions of individual helical structural elements without disruption of base-pairing interactions ^25,29^. We used the catalytic core domain of RNase P (PDB ID: 3DHS) ^31^ (see Methods) to generate the initial simulated data, called Benchmark 0 (BM0, Extended Data Fig. 6), at seven different levels of imposed Gaussian noise, 5, 10, 15, 20, 30, 40 and 50% of the maximum Z-height of the AFM topographic image (Extended Data Fig. 7). An initial structure was best fit to the AFM molecular surfaces via classical Langevin coarse-grained (CG) molecular dynamics ^32^ for efficient sampling over broad conformational space to search for the best fit to an experimental AFM image.

The impact of the topographic information from AFM images is illustrated by the residue dynamic cross-correlation maps (DCCM), where the AFM-restrained DCCM is only a subset of the free (unrestrained) DCCM (Extended Data Fig. 8a). Our method recapitulates 3D topological structures of RNase P conformers with the lowest RMSDs ranging from 2.97 to 6.04 Å relative to the ground-truth (GT) structure in the top cohort, depending on the applied noise level (Extended Data Fig. 8b). Within the practical limit of computing power, the efficiency of recapitulation depends heavily on initial input structure models. We tested HORNET on five additional types of RNA with very different topological structures derived using various methods (Fig. 3): RNA S142 (BM1) and 1076 (BM2) (Extended Data Fig. 9 and 10, and Extended Data Table 4), which were generated using FARFAR2 ^33^; an AFM-derived structure of the adenosylcobalamin riboswitch (BM3) ^34^ (Extended Data Fig. 11), and topological structures of group II intron (BM4) ^35^ and RNase P (BM5) ^36^ (Extended Data Fig. 12), both of which were derived from small angle X-ray scattering data ^37^ (Fig. 3a). These represent the majority of classes of RNA larger than 210 residues in the PDB database.

## Identifying top structural models using unsupervised ML

**Fig. 1.**
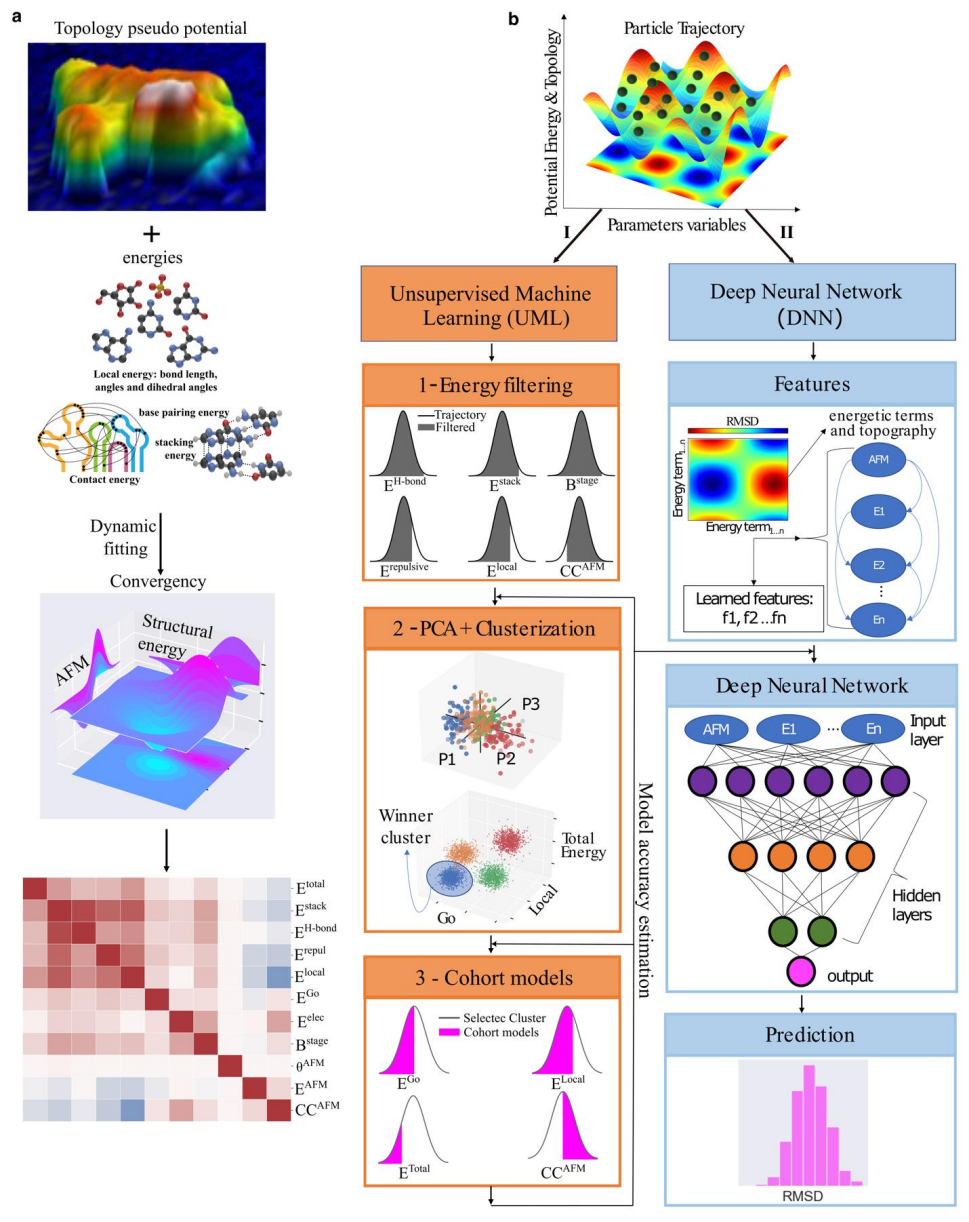
The workflow diagram of holistic RNA structure determination. **a,** Recapitulation of 3D RNA structures. Input: AFM-topography (x,y and z dimensions) and the native state information; dynamic fitting driven by the AFM and structure potentials; Output: All the generated models are evaluated based on individual energy values and the AFM topographic information. **b**, schematic illustration of the main steps for the top cohort selection and accuracy estimation using unsupervised ML (I) (analysis details is described in the Methods, Extended Data Fig. 1 and Extended Data Tables 1 and 2) and DNN (II) (analysis details is described in the Methods, Extended Data Fig. 2 and Extended Data Table 3).

Although top structures could be recapitulated together with vast numbers of other conformers, it is challenging to identify the top structures that are representative of the “true” structures underneath the AFM topographic surface using simple conventional correlation or approaches. The cross-correlation score $\left({CC}^{AFM}\right)$ between a molecular surface and a structure ^36^ alone is neither insufficient nor designed to identify top structure models because a near-perfect ${CC}^{AFM}$ could be achieved at the expense of structural integrity and the hierarchical folding principle. We used a holistic unsupervised ML by considering a combination of three types of information as input (Fig. 1b–I): (i) the energies associated with the primary chemical, secondary, and tertiary structures; (ii) ${CC}^{AFM}$ scores; and (iii) energy costs to account for the difference between the experimental and back-calculated AFM (pseudo-AFM) topographic image. Notably, all of the information is self-contained in the structure models and AFM topographic images, and none of it is presumptive.

An initial energy filtering (EF) to remove outliers from the full trajectory (see Methods, Extended Data Figs. 13–17) is followed by two steps of unsupervised ML including a principal component analysis (PCA) of all energy terms (Fig. 3a), and a successive clusterization algorithm that identifies the cluster of models with the lowest energetic distribution (Fig. 3b) according to the Go and total energies ^32^ (see Methods). The Go potential contains information about how well a given model folds. Importantly, the use of the Go energy does not prevent the AFM-biasing pseudo potential from sampling structures that are dramatically different from the initial structure. In the final step, the top cohort of models that exhibit both the lowest energies (Go, local, and total), as well as the best agreement with ${CC}^{AFM}$, are selected from the cluster (Fig. 3d, see Methods). Each step of the unsupervised ML pipeline iteratively selects the sub-population of the lowest RMSD models at each noise level (Fig. 3e and Extended Data Fig. 18). In all cases at the tested noise levels, almost all the top models fell within the final cohort, and the top 10 lowest-total-energy models from the unsupervised ML cohort had an average RMSD of 5 Å relative to the GT structure, with the lowest RMSD of ~3.5 Å. Interestingly, even at the higher noise levels, the average RMSD of the top 10 selected models was ~5 Å (Extended Data Table 5 and Extended Data Fig. 18), illustrating the robustness of HORNET.

## Estimating model accuracy using supervised ML

While unsupervised ML is capable of selecting top cohorts, it does not provide an estimate of accuracy, which is critical for the determination of unknown structures. Since the information about the accuracy of a conformer structure is contained within structure models as well as AFM topographic images, in principle, a well-trained DNN is capable of providing accuracy estimation, given the latest success in protein structure prediction ^3^. However, using DNN to estimate the accuracy of RNA structure models is far more challenging than for proteins ^16^. The DNN training models used for protein structure prediction ^3^ leverage the abundant information available in databases and the sequence-3D structure correlation and thus are not applicable to RNA because of the lack of both ^38^. Geometric deep learning has some success in structure prediction and accuracy estimates for relatively small RNAs but fails for large RNAs because of the lack of global restraints ^16^ and geometric equivalence among conformers, and thus is not applicable to predicting heterogeneous structures of the same sequence.

To overcome this problem, we created a pseudo-structure database containing more than three million conformer structure models, ~1.5 million trajectory structural models from BM0 and ~1 million each from BM1 and BM2 (see Methods). We assume this pseudo structure database to cover a broad conformational space between initial and final structure models that differ by as large as 35 Å in terms of RMSD. This input data taken from the pseudo database for the DNN training was partitioned as 80% for training the model (training set) and 20% for initial validation and testing of underfitting and overfitting in regularization processes (Extended Data Table 3). Aiming to assess if the learning by DNN is generalizable for not only the structural models from different trajectories but also from completely different RNAs ^39–41^, we use 5% of the dataset from BM5 as our validation dataset and BM3 and BM4 as our blind tests. Such a generalization scheme is shown to have a more realistic and robust assessment of the performance than that including fractions of data from all benchmark datasets ^40^. To assess the convergence of the trained models, we evaluated the progression of the loss function by the training epoch and selected the trained model and epoch that have the smallest loss in the validation set (Fig. 4a–b). Importantly, the input of our algorithm does not utilize information from structural databases. The DNN showed high performance for the predicted models in the initial training test set, with a Pearson coefficient of 0.97 (Fig. 4a). To evaluate the performance of HORNET for different trajectories and RNAs with different shapes, sizes, and sequences, we then tested the full trajectory of the five benchmarks to illustrate that our holistic DNN is learning generalizable structural “metrics” of accuracy, not merely memorizing specific structural features (Fig. 4c–g). Critically, all the benchmark tests used only data that the holistic DNN had never seen in either the training or initial validation. Our SML method (Fig. 1b–II) corroborates and cross-validates the results using unsupervised ML (Fig. 4h; Extended Data Table 6), while also estimating the accuracy of top cohorts in terms of RMSD. The tests in the five benchmark cases indicate that HORNET can recapitulate topological structure models from AFM images with an accuracy better than 6 Å RMSD.

## Validation using independent datasets

We evaluated a scenario where the initial structures were generated using FARFAR2 ^33^ and chosen based on their highest ARES ^16^ scores 9.23 and 9.04 for BM1 and BM2, respectively. The RMSDs between the GT structure and BM1 and BM2 are 14.3 Å and 20.0 Å, respectively and between the S142 and 1076 is ~18.7 Å, indicating that they adopt dramatically different topologies from each other and from the GT structure (Fig. 3a and Extended Data Fig. 10). In each of these cases, both unsupervised ML and DNN were capable of identifying the top cohorts (Fig. 3d and Fig. 4b,c,g). DNN performed well in the structure predictions, with Pearson correlations of 0.80 and 0.89, respectively (Fig. 4b,c). Furthermore, a poor RMSD predicted by HORNET is indicative of the non-convergence of a structure calculation. Structure S257, which has the best ARES score of 7.72 among 8000 conformers generated by FARFAR2 did not converge through dynamic fitting within practical computing time based on the poor predicted RMSD (Extended Data Fig. 19). Thus, S257 serves as an extreme case for testing our method, where the HORNERT was able to evaluate the non-convergency of the structure fitting (Extended Data Fig. 20) a primordial step in a real scenario where the GT structure is unknown and non-available. After a molecular dynamics simulation without AFM restraints starting from S257, we applied the unsupervised ML method without AFM-potential terms and picked the top five models from the cohort with the lowest total energies. These top five models used as initial models converged in a second round of dynamic fitting (Extended Data Figs. 21 and 22) where the DNN-predicted RMSD of the top structure is 3.6 Å (Extended Data Fig. 22b).

## Validation using data from a smaller RNA

The 210-nt cobalamin-sensing riboswitch (rCbl, BM3) is capable of folding into heterogeneous conformations ^2^. This RNA has a completely different sequence, shape, size, and folding from other RNAs used for training and validation in this study. Thus, BM3 serves as a unique benchmark for further testing the capability of HORNET. The crystal structure of BM3 was used ^42^ as the GT model and the initial structure is one of the conformers revealed by AFM ^34^, which has an RMSD of 10.2 Å from the GT structure. Our unsupervised ML was capable of identifying the top cohort of models with the lowest RMSD of ~2.7 Å, and the supervised ML was able to identify a similar cohort and correctly score to the top 10 models in terms of RMSDs with a Pearson score of 0.84 for the whole trajectory (Fig. 4d).

## Validation using data from structures generated from SAXS

BM4 and BM5 were chosen to demonstrate the capability of recapitulating structures where the initial models were generated using low-resolution experimental techniques. For this purpose, we used SAXS-derived models of BM4 (395 nts) and BM5 (298 nts) ^43^, whose initial models were 16.1 and 14.0 Å from their GT crystal structure models in terms of RMSD ^35,36^. During the dynamic fitting, both RNAs showed a wide energy landscape; however, unsupervised ML was able to select a cohort containing models with a minimum RMSD of 3.8 Å for BM4 and 5.4 Å for BM5 (Fig. 3). The SML performed well in scoring the models, especially in predicting the accuracy of the models of top cohorts with Pearson coefficients of 0.77 and 0.64 for BM4 and BM5, respectively (Fig. 4f,g). Considering the results from all benchmark tests, we conclude that HORNET is capable of estimating the accuracy of recapitulated structural models of the top selected cohorts in terms of RMSD. Predicted RMSD becomes less accurate beyond ~7.5 Å, which may be explained by both energetic and topographic equivalency among conformers, or the under-representative pseudo structure database (Fig. 4i).

## Determining structures of conformers using experimental AFM images

**Fig. 2|:**
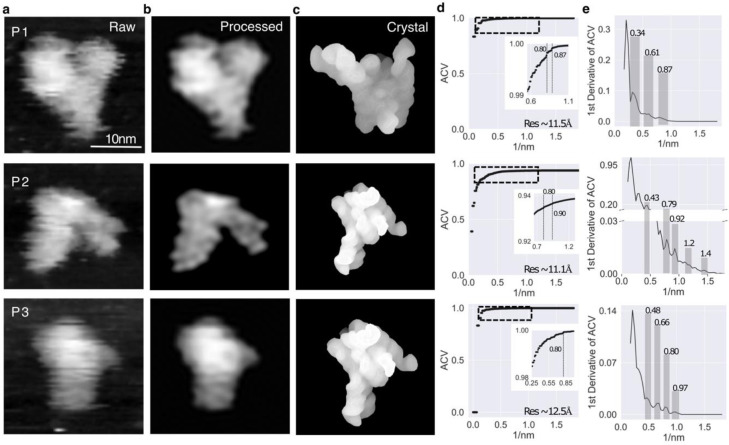
Images of three individual RNase P molecules in solution. **a,** Raw AFM image. **b,** processed AFM images with random noise removed. **c,** The crystal structure (pdb id 2a64) rendered in the molecular surface mode in similar orientations as the AFM particles. **d,** Resolution analysis of the AFM-topography, the auto-correlation value (ACV) of the AFM image of the particle. The spatial regimes with an abrupt decrease of ACV are shown in a zoom-in panel inset, where the kinks of ACV values indicate structure features present in the image at that spatial position, and the clear kinks for P1, P2 and P3 are highlighted by the dotted lines at ~0.87 nm^−1^(11.5Å), 0.87 nm^−1^(11.5Å), and 0.87 nm^−1^(11.5Å), respectively. **e,** The first derivative of the ACV plots to observe the full-profile variation. The gray areas indicate ACV discontinuity regimes, with the maximum spatial threshold at ~0.34 nm^−1^(29Å) and a minimum of ~ 0.87 nm^−1^(11.5Å), ~1.4 nm^−1^(7.2Å), ~0.97 nm^−1^(10.3Å), for P1, P2 and P3, respectively.

One ultimate goal is to recapitulate the structures from experimental AFM images of individual RNA molecules with unknown conformations (Figs. 2a and 5). The AFM images of the three particles showed discernable structural ^36^ features, and the layouts of the structural elements in each particle were distinctly different from one another and from the crystal structure, evidenced by their ${CC}^{AFM}$ scores, of 0.77, 0.80 and 0.87 for AFM particles P1, P2 and P3, respectively (Fig. 2C). P1 had the lowest ${CC}^{AFM}$ score, suggesting a significant deviation from the crystal structure. Thus, these three particle images capture three different conformations along the folding landscape (Fig. 2a). The dynamic fitting of the crystal structure for P1, P2 and P3 shows significantly different conformational landscapes (Fig. 5a and Extended Data Fig. 23). P1 samples a vast area of atomic displacements, while P3 had an intermediate sampling. P2 had the most restricted displacements, likely due to the presence of more short-distance information in the P2 AFM image (Fig. 2e) than the others. The 3D dynamic fitting trajectories were analyzed by unsupervised ML and DNN (Extended Data Tables 7 and 8). The best-recapitulated models for P1, P2 and P3 were between 4–6 Å RMSD (Fig. 5b and Extended Data Table 7). The unsupervised ML and DNN select models in similar ranges of energies and ${CC}^{AFM}$ (Fig. 5c, Extended Data Table 8). The DNN distributions to smaller values, with a minimum RMSD of 4.4 and 4.7, respectively (Fig. 5c). This shows that the trained model is capable of identifying and scoring different ranges of atomic displacements. The RMSDs between the top recapitulated model for each particle and crystal structure, excluding residues with missing electron density in the crystal structure were ~15, ~11, and ~7 Å, for P1, P2, and P3, respectively. *Rg* of the crystal structure is 45.1 Å, indicating a more compact structure, similar to that of the P3 conformer (45.8 Å). Indeed, P3 most resembles the crystal structure by visual inspection (Fig. 5b). On the other hand, P2 appears to be more extended and the recapitulated structures shows that major fluctuations occur in the regions where the crystal structure could not be modeled due to insufficient electron density (Figs. 5d and 2a,c).

## Discussion

Our method addresses major challenges in studying topological structures of highly heterogeneous and flexible RNA molecules by obviating the dependency on signal-averaging. The ability to recapitulate topological structures from AFM images of individual RNA molecules could drastically expand our knowledge of the heretofore uncharted RNA 3D conformational space ^10^, far beyond the few snapshots of static structures in databases. Given that more than 80% of the human genome is transcribed into RNA ^44^, more than 80% of which contain structural elements, HORNET has the potential to accelerate our understanding of the topological structures of large RNA with known biological significance.

Estimating the accuracy of an unknown structure is a grand challenge in structural biology ^45^. Recent success in protein ^3^ and progress in RNA structure prediction ^16^ is highly encouraging. Because of the conformational heterogeneity of RNA, incorporating individual conformer-specific topographic global restraints for the recapitulation and accuracy evaluation of heterogeneous structure models using DNN is a viable approach to chart the highly heterogeneous RNA conformational space. Given a sufficient structure database that covers a full RNA conformational space for DNN training and topographic information, we believe that accurate topological structures of individual RNA conformers in solution can be determined.

## Methods

### RNA structure calculation applying topographic restraint

Given that duplexes are well-conserved and the predominant building blocks in folded RNA structures by far ^23^, they could be considered semi-rigid bodies within a folded RNA structure. Since they are covalently connected, these duplexes can be treated as kinematic chains. Adding kinematic constraints between rigid bodies will significantly decrease the degrees of freedom of a rigid body system ^46^, and imposing the topographic constraints in addition to the kinematic constraints further reduces the degrees of freedom of sampling space.

A high-resolution AFM image is more than just a “frame” of a molecule. The width and pitch of an A-form RNA duplex are ~25 and 30 Å, respectively, which are on a similar scale to a sharp AFM probe and sensitive to detection. Thus, given an achievable imaging resolution of 10–15 Å^21^ (Fig. 2), major structural features such as grooves and pitches of long duplexes along with molecular shapes and topological folds of larger structured RNAs in solution are discernable in high-resolution AFM images. Thus, a high-resolution AFM image ^13,47–49^ of a molecule is a 3D frame with details about topographic information on individual molecules. The explicit expression of the physical relationship between a molecular structure and the topographic molecular surface is defined.

The AFM images of individual molecules are digitized into molecular surfaces Z(x,y), where Z is the height at the (x,y) position. A differentiable cross-correlation function $CC^{AFM}(x,y,z)$ (Eq. 1), the corresponding pseudopotential $V^{AFM}(x,y,z)$ (Eq. 2) and the backside potential $B^{\mathrm{stage}}(x,y,z)$ (Eq. 3) are developed and used for dynamic fitting ^32^.

(1) $$CC^{AFM}(x,y,z)=\frac{\sum_{i}I_{i}^{\exp\;}I_{i}^{\mathrm{sim}}(x,y,z)}{\sqrt{\sum_{i}{\left(I_{i}^{\exp\;}\right)}^{2}}\sqrt{\sum_{i}{\left(I_{i}^{\mathrm{sim}\;}(x,y,z)\right)}^{2}}}$$

(2) $$\begin{array}{c} V^{AFM}(x,y,z)=\theta^{AFM}Nk_{B}T[1-CC^{AFM}(x,y,z)] \end{array}$$

(3) $$B^{\mathrm{stage}}(x,y,z)=\sum_{i}^{N}4\varepsilon \left[{\left(\frac{d_{i}}{2z_{i}}\right)}^{12}-{\left(\frac{d_{i}}{2z_{i}}\right)}^{6}\right]$$

where $CC^{AFM}(x,y,z),I_{i}^{exp}$ and $I_{i}^{\mathrm{sim}\;}(x,y,z)$ are the cross-correlation, experimental and back-calculated heights, respectively, of the *i^th^* pixel at the (x,y) position on the molecular surface; $\theta^{AFM}$ is an empirical scaling factor of the AFM force potential $V^{AFM};N$ and $k_{B}$ are the total number of beads (for RNA, is the total of sugar, phosphate and bases) in the molecule and the *Boltzmann* constant, respectively; $B^{\mathrm{stage}}(x,y,z)$ is the backside information in the form of Lennard-Jones potential that parameterizes the interaction between the particles on the molecule’s backside and the mica surface; $z_{i}$ and $d_{i}$ are the Z position and interparticle distance of particle i, respectively, and ε is the interaction-energy factor. The algorithm for simulating the tip dilation effect^20^ is used with the set of empirically determined parameters. $B^{\mathrm{stage}}(x,y,z)$ ensures that the backside of a molecule is in contact with the mica surface, a mere reflection that the molecule must be immobilized to be imaged. Eq. 1 to 3 encapsulates the topological information of both the front and back of a molecule. Dynamic fitting is driven by $V^{AFM}(xyz)$ and $B^{\mathrm{stage}}(x,y,z)$, and it may achieve the apparent best fit to the molecular surface with a near-perfect correlation score at the expense of both the primary and secondary structures, resulting in a severely distorted RNA 3D structure underneath. To resolve this problem, energy terms, $E^{c}$(covalent energy) and $E^{nc}$ (non-covalent energy) that enforce the RNA hierarchical folding principle are applied together with the AFM pseudopotential (Eq. 1 to 3):

(4) $$E^{\mathrm{total}\;}=V^{AFM}(x,y,z)+\theta^{c}\sum \sum E^{c}+\sum \theta^{nc(i)}\sum E^{nc}$$

where total energy $E^{\mathrm{total}\;}$ is the system energy for the molecule; $\theta^{c}\sum \sum E^{c}$ is the covalent energy term that includes bond lengths, angles, and dihedrals:

(5) $$\theta^{c}\left(\sum_{j}E_{j}^{\mathrm{angle}\;}+\sum_{k}E_{k}^{\mathrm{length}\;}+\sum_{l}E_{l}^{\mathrm{dih}\;}\right)$$

whereas the noncovalent energy term $\sum \theta^{nc(i)}\sum E^{nc}$ includes stacking, base-pairing, short- and long-range interactions, specifically van der Waals and electrostatic interactions:

(6) $$\left(\theta^{\mathrm{stacking}\;}\sum_{m}E_{m}^{\mathrm{stacking}}+\theta^{\mathrm{pairing}\;}\sum_{n}E_{n}^{\mathrm{pairing}\;}+\theta^{\mathrm{contact}\;}\sum_{o}E_{o}^{\mathrm{contact}\;}\right)$$

$\theta^{\mathrm{AFM}},\theta^{c},\theta^{\mathrm{stacking}},\theta^{\mathrm{pairing}}$ and $\theta^{\text{contact}}$ are the scaling factors for AFM, covalent, and secondary structural interactions including stacking, base-pairing, and contacts, respectively. We usually set $\theta^{\mathrm{contact}}$ to unity to avoid bias towards the initial structure, whereas $\theta^{\mathrm{AFM}},\;\theta^{c},\;\theta^{\mathrm{stacking}}$ and $\theta^{\mathrm{pairing}}$ are empirically determined to achieve the optimal balance between enforcing the integrity of primary and secondary structures (the hierarchical principle) and achieving the best fit to the topological restraints at the same time (Eq. 1 to 3). In this study, we found that the optimal empirical values are $\theta^{c}=5,\theta^{\mathrm{stacking}}=9$ and $\theta^{\mathrm{pairing}}=9$. The optimal value for $\theta^{AFM}$ is dependent on the topography of the particle, the noise level of an image, and the closeness of the initial structural model to the “true” structure underneath the molecular surface. We scan through dynamic fitting calculations with $\theta^{\mathrm{AFM}}$ from 2 to 50.

In summary, the topological restraint imposed by Eq. 1 to 3 together with the energetics of the primary, secondary and tertiary structures significantly reduces the degrees of freedom that a molecule can sample in a coarse-grained MD trajectory. To perform the coarse-grained MD trajectory, we make used of Cafemol software. A user friendly pipeline and calculation setup is described in the Supporting Information material and code availably at https://github.com/PNAI-CSB-NCI-NIH/HORNET. The challenge now is to identify the structural models closest to the “true” structure underneath the AFM molecular surface.

### Unsupervised machine learning

Our UML approach assumes that the classical molecular dynamics simulation guided by topographic information can sample the real native conformational space of the RNA and that the correct models can be identified based on the established hierarchical folding principle ^50^, energetics ^51^ and agreement with topographic restraints. Our UML algorithm is able to decipher the underlining correlation of the data set, resulting in the recognition of generalizable models without pre-training or data labeling. Each analyzed data set (trajectory) is unique, and the machine does not have any expected pre-labeled output from a given input. Our UML algorithm consists of three main steps: (i) energy filtering, (ii) principal component analysis (PCA) and clustering, and (iii) cohort model selection.

### Energy filtering

This step is performed to remove outliers of unstable conformers generated in the trajectory. The input data is filtered from the whole trajectory (raw data) with m total frames (trajectory structures), and the filter function is applied based on the mean value of each j energy component:

(7) $$\langle E_{j}\rangle =\left(\sum_{i=1}^{m}\;\;E_{j}\right)/m$$

where j represents the seven components used: $E^{\mathrm{repulsive}},\;E^{\mathrm{local}},\;E^{\mathrm{stacking}},\;E^{\mathrm{pairing}},\;B^{\mathrm{stage}},CC^{\mathrm{AFM}}$ and $E^{\mathrm{total}}$. The frames are filtered by selecting low values of the energy components based on the number of sigma (nσ) cutoff, where n is empirically determined after extensively testing over different simulated data (benchmark 0):

(8) $$n\sigma_{E_{j}}=\frac{E_{j}-\left\langle E_{j}\right\rangle }{\sigma_{E_{j}}}$$

All variables are normalized using Eq. 8 and each component now has a mean value centered at zero and described as standard units of σ^52^. The filtering analysis is performed in two steps. First the $n\sigma_{E_{j}}$ threshold is applied to remove outliers from the raw data for the five individual energy components: $E^{\mathrm{repulsive}},\;E^{\mathrm{local}},\;E^{\mathrm{stacking}},\;E^{\mathrm{pairing}},\;B^{\mathrm{stage}}$, followed by filtering using ${CC}^{AFM}$ and $E^{\mathrm{total}}$ cutoffs. The cutoff limits of $n\sigma_{E_{j}}$ are described in (Extended Data Table 1).

### Principal component analysis (PCA) and clustering

There are a total of 10 features which include energetic and topographic information that each frame is associated with. We reduce data dimensionality to a small number of components while maintaining the maximum amount of statistical information by applying PCA and clustering. These 10-dimensional vectors are the features describing energetics and topology, the i-th element of the m-th structure $\left\{l_{m}^{i}\right\}$:

(9) $$l=\sum_{m}^{i}l_{m}^{i}$$

The vector I has index m that runs from 1 (first frame) to m (last frame) representing all the structure models of the trajectory and all features, which include energy terms $E^{\mathrm{total}},\;E^{Go},\;E^{\mathrm{local}},\;E^{\mathrm{stacking}},\;E^{\mathrm{pairing}},\;E^{\mathrm{repulsive}},\;E^{\mathrm{electrostatic}},\;B^{\mathrm{stage}}$ and $V^{AFM}$, and $CC^{AFM}$, after the initial filtering. All $l_{1}^{1}\ldots l_{m}^{10}$ vectors could be represented by a data matrix R(m,10). For example, $a_{m,i}\;m$ is the frame of the trajectory and i is the energy/topology information:

$$R=\left[\begin{array}{ccc} a_{1,1} & \cdots & a_{1,10}\\ \vdots & \ddots & \vdots \\ a_{m,1} & \cdots & a_{m,10} \end{array}\right]=\left[\begin{array}{cccccccccc} E_{total}^{1} & V_{AFM}^{1} & E_{local}^{1} & E_{stacking}^{1} & E_{repulsive}^{1} & E_{pairing}^{1} & E_{electrostatic}^{1} & B_{stage}^{1} & E_{Go}^{1} & CC^{1}\\ \vdots & \vdots & \vdots & \vdots & \vdots & \vdots & \vdots & \vdots & \vdots & \vdots \\ E_{total}^{m} & V_{AFM}^{m} & E_{local}^{m} & E_{stacking}^{m} & E_{repulsive}^{m} & E_{pairing}^{m} & E_{electostactic}^{m} & B_{stage}^{m} & E_{Go}^{m} & CC^{m} \end{array}\right]$$

To derive a linear combination of the 10 features and maximize variance among the components we applied principal component analysis (PCA). In this procedure, we describe our matrix R with another linear combination basis. In other words, we can find a transpose vector $\zeta^{\prime }$ of constants $\zeta_{1},\zeta_{2},\ldots ,\zeta_{10}$ where:

(10) $$\zeta_{1}^{\prime }R=\zeta_{1}l_{1}+\zeta_{2}l_{1}+\zeta_{3}l_{m}+\cdots +\zeta_{10}l_{1}+\cdots +\zeta_{10}l_{m}$$

However, the Eq. 10 is not a unique solution to describe a linear combination basis of the matrix R, other non-correlated solution $\zeta_{2}^{\prime }R$ exists, and continues up to the $n^{\mathrm{th}}$ linear function with $\zeta_{n}^{\prime }$:

(11) ζR=Y

with ζ the n-rows of the linear transformation matrix ζ that represents the principal components (PC), Y is the matrix (m×n) related product of ζR. The variance presented in this linear combination is quantified using the covariance matrix S ^53^:

(12) $$S_{kj}=\frac{1}{N}\sum_{m=1}^{N}\left(E_{k}^{m}-\left\langle E_{k}\right\rangle \right)\left(E_{j}^{m}-\left\langle E_{j}\right\rangle \right)$$

Where k and j are combinations of the terms of I (Eq.9). In order to have linear combinations and uncorrelated variables, the covariance matrix needs to be transformed to a diagonal matrix A, that is:

(13) $$P^{T}SP=A$$

P is orthogonal and can be described as a normalized matrix, $P^{T}$ denotes the transpose of P. This last equation can be resolved by calculating the eigenvector of the covariance matrix: Sx=λx, where x is a vector of P with eigenvalues λ, which are the diagonal elements of A. In our analysis, this equation is resolved using singular value decomposition (SVD)^54^. Before applying SVD, we standardize all the features using Eq. 8.

To define the number of components describing the trajectory, a primary screening is performed for each dataset to analyze the cumulative variance *versus* the number of components. The general profile for this plot can be seen in Extended Data Fig. 1a, and the initial values show a steep increase that reaches a very low variance rate with a flat area for a higher number of components. The chosen cutoff is located at the beginning of the lower cumulative variation rate. Using the PCA-space data matrix the k-means algorithm ^55^ is applied to separate the data into clusters where each population is defined by the minimalization of the inter-cluster entity distances to the geometric center. A similar strategy is used to define the number of clusters for each trajectory, but now the threshold is defined by the correlation plot between the within-cluster sum of squares (WCSS) and the number of clusters. The number of clusters is decided using the first derivative of the WCSS (Extended Data Fig. 1b), at the intersection between considerable variations and a flatter pattern. The outcome is that the representative cluster with the correct structure population should be the one with the structures containing the lowest native energy values. Using that criterion, the $E^{\mathrm{local}},\;E^{\mathrm{total}}$ and $E^{Go}$ energy distributions are analyzed and the cluster with lower mean distribution is selected.

### Cohort model selection

The cohort of models from the trajectory is selected using the representative cluster. The final selection criteria are applied assuming that the best models must be observed at the highest cross-correlation with the AFM topography $\left(CC^{AFM}\right)$ and, at the same time, the energy values need to be populated at the lowest values. A threshold filtering procedure is applied to the representative cluster using the cutoff values described in Extended Data Table 2.

### Supervised deep neural network design

Based on the question of whether the most fundamental characteristics of models such as their energetics and known topology of a structure contained in the AFM experimental data would provide enough information to consistently determine the RMSD between the structural model and an unknown GT structure, we designed a deep neural network (DNN)^56^ to learn how these fundamental characteristics could be intrinsically correlated.

### Data Preparation and Features

The dense layers of our DNN are connected by passing the information of each layer forward to the next one, known as a feedforward neural network. The training of a DNN relies on the ability to find the best weights $\left(W_{j}\right)$ and bias term $\left(b_{j}\right)$ in all the layers in a way that minimizes the difference between the true value of the feature to be predicted and the output layer $\left(A_{L}\right)$ of this DNN:

$$A_{1}=g\left(W_{1}^{T}\cdot X+b_{1}\right)\;A_{2}=g\left(W_{2}^{T}\cdot A_{1}+b_{2}\right)\;\;\vdots \;A_{L}=g\left(W_{L}^{T}\cdot A_{L-1}+b_{L}\right)$$

where $W_{1},W_{2},\ldots W_{L}$ are the weight matrices for the layers 1,2,…L, respectively, containing all the learning weights per feature and per neuron stacked together $\left(n\times k_{l}\right)$, where n is the number of features and $k_{l}$ is the number of neurons in the *l*^th^ layer, while $b_{l}$ is the learning bias term at the *l*^th^ layer, one per neuron $\left(1\times k_{l}\right)$. The function g is the activation function for this layer, and $A_{l}$ is the result of the activation over the operation on the *l*^th^ layer, which is passed forward to the next layer. X is a matrix of size n×m, where n is the number of features and m is the number of training examples.

In our case, the data X was prepared using only the energetic and topographic information. We added a single pre-defined new feature that improved the model convergence and accuracy, and most of the features selected to train the model are those already used in UML. These include energy terms, such as $E^{\mathrm{total}},\;E^{\mathrm{local}},\;E^{go},\;E^{\mathrm{repulsive}},\;E^{\mathrm{stacking}},\;E^{\mathrm{pairing}},\;E^{\mathrm{eletrostatic}}$, and topographic constraint AFM potential $V^{AFM}$ and cross-correlation $CC^{AFM}$. The feature created as a combination of other features is:

$$•\;CC^{AFM}\text{to}\;\text{the}\;\text{power}\;\text{of}\;N\times \;\text{total}\;\text{energy}:C{C^{AFM}}^{N}\times E^{\mathrm{total}}$$

where N=7 is empirically determined to increase the weight of top $CC^{AFM}$ as a global restrain. For a generalized model across RNA’s with different sizes, the energies $E^{\mathrm{total}},\;E^{\mathrm{local}},\;E^{\mathrm{go}},\;E^{\mathrm{repulsive}},\;E^{\mathrm{eletrostatic}}$ and the potential $V^{AFM}$ are normalized by the number of nucleotides of the RNA. The energies $E^{\mathrm{stacking}}$ and $E^{\mathrm{pairing}}$ are normalized by the number of base-stacking and base-pair interactions. $V^{AFM}$ is further normalized by $\theta^{AFM}$, while $E^{\mathrm{stacking}}$ and $E^{\mathrm{pairing}}$ were normalized by $\theta^{\mathrm{stacking}}=9$ and $\theta^{\mathrm{pairing}}=9$, and $E^{\mathrm{local}}$ by $\theta^{c}=5$. After the featurization and normalization, we applied a standard scaling so that the distribution used for training (and testing) would be standardized to allow an optimized convergence of the training. Consequently, all the data used for subsequent RMSD evaluations need to be normalized by the same training normalization parameters to be consistent with the training data and evaluation.

In this study, the output of the DNN is driven to be as close as possible to the RMSD of the training examples by using all the discussed features. The weights and bias for the first layer $\left(W_{1},b_{1}\right)$ with k neurons will be:

(14) $$W_{1}=\left[\begin{array}{ccccc} w_{1}^{E^{\mathrm{total}}} & w_{2}^{E^{\mathrm{total}}} & \cdots & w_{k-1}^{E^{\mathrm{total}}} & w_{k}^{E^{\mathrm{total}}}\\ w_{1}^{E^{\mathrm{local}}} & w_{2}^{E^{\mathrm{local}}} & \cdots & w_{k-1}^{E^{\mathrm{local}}} & w_{k}^{E^{\mathrm{local}}}\\ w_{1}^{E^{go}} & w_{2}^{E^{go}} & \cdots & w_{k-1}^{E^{go}} & w_{k}^{E^{go}}\\ \vdots & \vdots & \vdots & \vdots & \vdots \\ w_{1}^{E^{\mathrm{pairing}}} & w_{2}^{E^{\mathrm{pairing}}} & \cdots & w_{k-1}^{E^{\mathrm{pairing}}} & w_{k}^{E^{\mathrm{pairing}}}\\ w_{1}^{V^{AFM}} & w_{2}^{V^{AFM}} & \cdots & w_{k-1}^{V^{AFM}} & w_{k}^{V^{AFM}}\\ w_{1}^{CC^{AFM}} & w_{2}^{CC^{AFM}} & \cdots & w_{k-1}^{CC^{AFM}} & w_{k}^{CC^{AFM}} \end{array}\right],b_{1}=\left[\begin{array}{c} b_{1}\\ {\;b}_{2}\\ \vdots \\ b_{k-1}\\ {\;b}_{k} \end{array}\right]$$

where each column represents the weights for a single neuron and each row the weight per feature, while b is the bias term, one per neuron. The data X and the target of the RMSD prediction on the output layer $\left(\overset{ˆ}{y}\right)$, on the other hand, would be:

(15) $$X=\left[\begin{array}{ccccc} x_{1}^{E^{\mathrm{total}}} & x_{2}^{E^{\mathrm{total}}} & \cdots & x_{m-1}^{E^{\mathrm{total}}} & x_{m}^{E^{\mathrm{total}}}\\ x_{1}^{E^{\mathrm{local}}} & x_{2}^{E^{\mathrm{local}}} & \cdots & x_{m-1}^{E^{\mathrm{local}}} & x_{m}^{E^{\mathrm{local}}}\\ x_{1}^{E^{\mathrm{go}}} & x_{2}^{E^{\mathrm{go}}} & \cdots & x_{m-1}^{E^{\mathrm{go}}} & x_{m}^{E^{go}}\\ \vdots & \vdots & \vdots & \vdots & \vdots \\ x_{1}^{E^{\mathrm{pairing}}} & x_{2}^{E^{\mathrm{pairing}}} & \cdots & x_{m-1}^{E^{\mathrm{pairing}}} & x_{m}^{E^{\mathrm{pairing}}}\\ x_{1}^{V^{AFM}} & x_{2}^{V^{AFM}} & \cdots & x_{m-1}^{V^{AFM}} & x_{m}^{V^{AFM}}\\ x_{1}^{CC^{AFM}} & x_{2}^{CC^{AFM}} & \cdots & x_{m-1}^{CC^{AFM}} & x_{m}^{CC^{AFM}} \end{array}\right],\overset{ˆ}{y}\to y=\left[\begin{array}{c} x_{1}^{RMSD}\\ x_{2}^{RMSD}\\ \vdots \\ x_{m-1}^{RMD}\\ x_{m}^{RMSD} \end{array}\right]$$

where the columns in X represent each training example (up to m examples) and the rows of each feature. The output layer should predict values for the RMSD as closely as possible to the real ones for all the data examples.

### Loss function

The process of learning in an artificial neural network (ANN) depends on the loss function. The mean squared error (MSE, also known as L2 loss or quadratic loss) and the Huber loss, had both great performances in our training:

(16) $$\;L_{MSE}=\sum_{i=1}^{m}\;{\left({\overset{ˆ}{y}}_{i}-y_{i}\right)}^{2}\;L_{\mathrm{Huber}\;}=\sum_{i=1}^{m}\;\;\left[\begin{array}{cc} \frac{1}{2}{\left({\overset{ˆ}{y}}_{i}-y_{i}\right)}^{2} & \;\mathrm{for}\;\left|{\overset{ˆ}{y}}_{i}-y_{i}\right|<\delta \\ \delta \cdot \left(\left|{\overset{ˆ}{y}}_{i}-y_{i}\right|-\frac{1}{2}\delta \right) & \;\mathrm{for}\;\left|{\overset{ˆ}{y}}_{i}-y_{i}\right|\geq \delta \end{array}\right.$$

where $\overset{ˆ}{y}$ is the prediction for a single training sample and y its true value. δ in the Huber loss sets the region where the loss will assume a squared difference or absolute difference, so it does neither overweight the outliers such as in the MSE loss, nor simplify the loss by the averages. In the training of our DNN model, we used the Adam^57,58^ optimizer to minimize the loss function.

### Underfitting and Overfitting

To avoid overfitting and to be able to keep increasing the complexity of our ANN we added regularization penalties to the training. Within the known regularizers we evaluated training using Ridge regression (L2 regularizer) and dropout technique. Ridge regression adds a penalty to the loss function term for all the weights squared, preventing the weights to assume too higher values. In the dropout technique, in each step of the training/optimization, some neurons have a given (set) chance to be turned off. We also tested increasing the size and variety of the dataset by adding more data (trajectories) - see Extended Data Table 3.

### Optimized architecture

To train the DNN and access its performance, we split dataset X (which contains only one kind of RNA) into 2 parts: the training, and the initial training test set, where we could check the regularization effect over the same trajectory and assure that the regularization was blocking the train to overfit the trajectory over the split, hence providing similar loss on both sets. The training set had 80% of the m data examples, while this initial training test set had 20%. The optimized dataset that yields the best performance was built using the whole data from the BM 0 data, with an addition of 5% of data from trajectories of the BM1 and BM2 each (Extended Data Fig. 2). The validation set was created by using a different RNA trajectory simulation, BM5, so that the best loss on the validation set would point to the place where the training and learning with a given RNA was still generalized to another RNA or trajectory, applying early stopping on the evaluated loss considering RMSDs up to 10 Å to weight a better performance on smaller RMSDs than larger ones. Hence, the validation set was used for both tuning the hyperparameters and for selecting the best-trained model, while further tests over the benchmarks address if our model can generalize its findings and learnings to other RNAs not contained in the training data, with different RNA sizes, assessing what would be the real performance of our model to other unknown RNAs and trajectories than the one used for training.

We optimized the architecture for this work by many step-by-step random searches and subsequent fine-tuning of the hyperparameters, which include the number of layers, the number of neurons per layer, weight initialization, neuron activations, regularization penalties and types, the optimizer algorithm as well as the learning rate. Additionally, more than 50 different compositions of the training dataset were also used for training models (Extended Data Table 3). The number of hidden layers tested (also by a random search) was between 1 and 10 hidden layers. The number of neurons in each layer, on the other hand, was tested basically in 3 types: (i) starting with a high number of neurons in the first layer and decreasing this as the number of layers increases; (ii) starting with a medium number of neurons in the first layer, and increasing the number of neurons on the next layers until reaching the middle layer, then decreasing as we continue to the last layer, and (iii) through a random search, where the number of neurons per layer was picked randomly as a multiple of 8, being able to assume values from 8 to 256 neurons per layer. For architectures with 5 or more layers we included batch normalization within layers.

The non-linear activations tested were relu, leaky-relu, elu and gelu for each layer separately, or a selu^59^ activation set for all layers. For regularization, each layer could use either the Ridge regression and/or a Dropout^60^ chance (for selu the Alpha Dropout^59^ was used instead of Dropout to keep the self-normalizing properties). Our optimized architecture has only 3 hidden layers with a decreasing number of neurons, 128 in the first layer, 64 in the second, and 16 in the third, using elu activation with a common dropout rate of around 20% as the regularizing agent. Deeper networks also had a good performance, but with the cost of many weights to train without clear improvement. The total number of trainable parameters with the current architecture is around 11k. Within initializations, we tested Glorot uniform, Lecun normal and He normal, with the latest achieving the best performance as the weight initializer and using Adam as the optimizer algorithm with a standard learning rate of 0.001, with the mini-batch size of 128 and using Huber loss.

Extended Data Table 3 summarizes all the hyperparameters for searching and tuning, as well as different datasets and data filters applied to create dataset X as input to the models. The models were trained using NIH-HPC (Biowulf) k80/k100x nodes:

- K100x node: 36 × 2.3 GHz (Intel Gold 6140), hyperthreading, 25 MB secondary cache, 4 × NVIDIA V100-SXM2 GPUs (32 GB VRAM, 5120 cores, 640 Tensor cores).
- K80 node: 28 × 2.4 GHz (Intel E5-2680v4), hyperthreading, 35MB secondary cache, 2 × NVIDIA K80 GPUs with 2 × GK210 GPUs each (24 GB VRAM, 4992 cores)

### RNA sample preparation

The RNase P RNA was prepared by following the previously published procedure^36^. Briefly, the RNase P RNA was transcribed *in vitro* with recombinant T7 phage RNA polymerase from a double-strand DNA template that was amplified by PCR from linearized DNA plasmid, which encodes a full-length RNase P RNA from *B. Stearothermophilus* with an upstream T7 RNA polymerase promoter. Transcribed RNA was purified by denaturing polyacrylamide gel electrophoresis (PAGE) containing tris-borate with EDTA (TBE) and 8M urea. The RNA was excised and eluted from the gel in RNA elution buffer (300 mM Sodium acetate pH 5.3, 0.1 mM EDTA) for 12 hours at 4 °C. The eluted RNA was filtered using a 0.2 mm Ultrafree-MC centrifugal filter device (Millipore). Purified RNA was subjected to several buffer exchanges using a Centricon unit (Millipore) with 30kDa molecular weight cut-off membrane against refolding buffer (50 mM MES buffer pH6.8, 100 mM KCl, 1 mM MgCl_2_), then concentrated to 2μM, aliquoted, and stored at −80 °C before utilization.

For AFM experiments, the RNA sample at 2μM concentration was annealed in the refolding buffer (50 mM MES buffer pH6.8, 100 mM KCl, 10 mM MgCl_2_) at 65°C for 2 min followed by stepwise cooling to 37°C over 30 min, and then kept at 4°C before AFM measurements. To dilute the RNA sample to the required concentration (20 nM) for AFM, 1:100 volume of low-salt buffer (50 mM MES buffer pH6.8, 10 mM KCl, 1 mM MgCl_2_ (preequilibrated at 4°C) was used, and the diluted sample was immediately deposited onto mica pre-treated with 1-(3-aminopropyl) silatrane (APS) for immobilization^61^.

### Experimental AFM topography

#### Experimental AFM image acquisition

The detailed procedure for the AFM image acquisition is described elsewhere^34^. Here is a brief outline of the procedure. P1, P2 and P3 particle images (Extended Data Fig. 3) were recorded under the solution conditions described above using a Cypher VRS AFM (Asylum Research, Oxford Instrument) at 4°C with amplitude-modulated AC mode at a scan rate of 1 Hz (commonly known as tapping mode) using FASTSCAN-D-SS probes (Bruker, CA). For RNA immobilization, 50 mM APS stock was freshly diluted 300-fold in deionized water right before use and then used to coat a freshly cleaved muscovite mica (Grade V1) (Ted Pella Redding, CA) and incubated for 30 min, followed by rinsing the mica surface with deionized water and drying gently with filtered nitrogen gas.

#### Image processing

The detailed procedure for AFM image processing is described elsewhere^34^. Briefly, raw images were first processed using SPIP (Scanning Probe Image Processor) software: plane leveling to the particle-free region by applying 3^rd^-order polynomial, followed by spike filtering to remove artifact streaks, and FFT (Fast Fourier Transform) to remove high-frequency noise (Extended Data Fig. 3). The final image resolution was increased to 4096×4096 pixels by doubling the number of pixels twice. Single-particle images were cropped from the processed images and converted to pseudo-AFM (*.txt) with a digital resolution of 5-Å per pixel in MountainsSPIP software for structure calculation.

### AFM noise estimation

For quantification of the noise present in the Z coordinate, we used the cropped single molecule from the full recorded AFM topography as input, and the Z values were collected for defined x - and y-coordinates of the “*empty*” area around the molecule. The Z-coordinate values of the empty horizontal and vertical spaces can be described by a normal function, where the mean value of this distribution represents the mean noise and the uncertainty as the standard deviation (sigma). The mean noise value and uncertainty were evaluated for P1, P2 and P3 before and after image processing (Extended Data Fig. 4). In this analysis, we are considering all the experimental sources that result in noise randomly distributed over all recorded data as a background signal.

### AFM resolution estimation

The topography resolution assessment was performed using an auto-correlation value (ACV) approach^21^. There are two principal steps to be performed in this method. First, using the processed image, we calculate the 2D Fast Fourier transform (FFT) of the AFM image and a defined ring-size (pixels) cut-off is applied to select a portion of the image in Fourier space. Afterward, the image is back-calculated to real space; this step is described as a low-pass filtered Fourier ring. In the second step, we calculate the ACV between the original image (R) and the resulting one from the Inverse Fast Fourier Transform (IFFT) for each of the low-pass filtered rings $\left(R^{\prime }\right)$. The comparison between the original image with its resulting image from the low-pass filter is performed using the auto-correlation equation (Eq.17). A loop interaction was applied starting from low to high frequency, where the ACV starts from low correlation values up to values near to 1.0 where the low-pass cut-off is close to the particle dimension in real space. In Extended Data Fig. 5 we demonstrate some intermediate steps of the Fourier ring filter applied to P1, P2 and P3 particles. The ACV profiles show some discontinuities, or *kinks*, that are signatures of topographical features. To be able to distinguish these details, the first derivative of the ACV profile was calculated and shown in Fig. 2.

(17) $$ACV=\frac{\sum_{i}\left(R_{i}-\overset{‾}{R}\right)\times (R_{i}^{\prime }-\overset{-}{R^{\prime }})}{{\left\{\sum_{i}{\left(R_{i}-\overset{‾}{R}\right)}^{2}\times \sum_{i}{\left(R_{i}^{\prime }-\overset{-}{R^{\prime }}\right)}^{2}\right\}}^{1/2}}$$

where i is the (x,y) pixel position with a Z-height (R) of the original reference image and a Z-height (R’) after applying a low-pass filtered Fourier ring; $\overset{‾}{R}$ and $\overset{-}{R^{\prime }}$ represent the mean values of the Z-heights of reference and low-pass filtered images, respectively.

### Benchmarks design information

#### Benchmark 0

The initial structural model as Benchmark 0 was selected based on the best ARES score of 9.9^16^ (Extended Data Fig. 6) from a pool of models calculated using Cafemol^32^. The RMSD between this structure and the ground-truth (GT) structure is 21.4 Å. The GT structure was generated based on the crystal structure of the catalytical domain of RNase P RNA, denoted as trRNaseP RNA^31^ (accession code: 3dhs). The missing electron densities of the structure were modeled using SimRNA^62^ and further refinement using Coot^63^.

AFM images of the GT model were calculated using a resolution of 5.0 Å per pixel, added with seven different simulated Gaussian noise levels, i.e., 5, 10, 15, 20, 30, 40 and 50% of maximum Z-height (Extended Data Fig. 7).

The dynamic fitting using k158597 as initial and the AFM topography of the GT was performed for all noise levels (Extended Data Fig. 7) using the common configuration described in Supplementary Information for a total of 20×10^6^ steps (~0.9μs).

#### Benchmarks 1 and 2

This benchmark was designed aiming to tackle unknown 3D structure models while the primary sequence and secondary structure information is known. For this task we first applied FARFAR2^64^ - rna_denovo application generating 8000 structure models of truncated RNase P RNA (trRNase P) using the primary sequence, secondary structure (Extended Data Fig. 9), and atom pair distance constraints of the well-known loop interaction L15.1- L5.1 described in detail previously^65^ (Extended Data Table 4). For structure refinement the minimize_rna function was applied as potential during the FARFAR2 structure prediction run, using parallel jobs on a 28-core 2.3 GHz x2695 processor.

The FARFAR2 scoring function was calculated for all the predicted models and analyzed as a function of the main energy terms. The sampled refined structures show a range of RMSD with a maximum of 46 Å and a minimum of 14 Å from the crystal model (PDB id 3dhs). We selected three models from all predicted structures from FARFAR2 using the following criteria: one model with the best ARES prediction (S257), one being located in the region of both ARES and FARFAR2 low scores and an RMSD from the GT structure of at least 20 Å (S1076), and one model with the lowest RMSD (S142), Extended Data Fig. 10.

ARES selected model S257 as the best model from the FARFAR2 ensemble, a model that presents dramatically different folds from the crystal model, and an RMSD of ~30 Å (Extended Data Fig. 10b). Using an RMSD threshold of 20 Å and scoring the models using the energetic scoring function of FARFAR2 and final score of ARES, the best model was S1076 with an RMSD of 20.0 Å, where this model shows a folding similar to the crystal model (Extended Data Fig. 10b).

#### Benchmark 3

**Fig. 3|:**
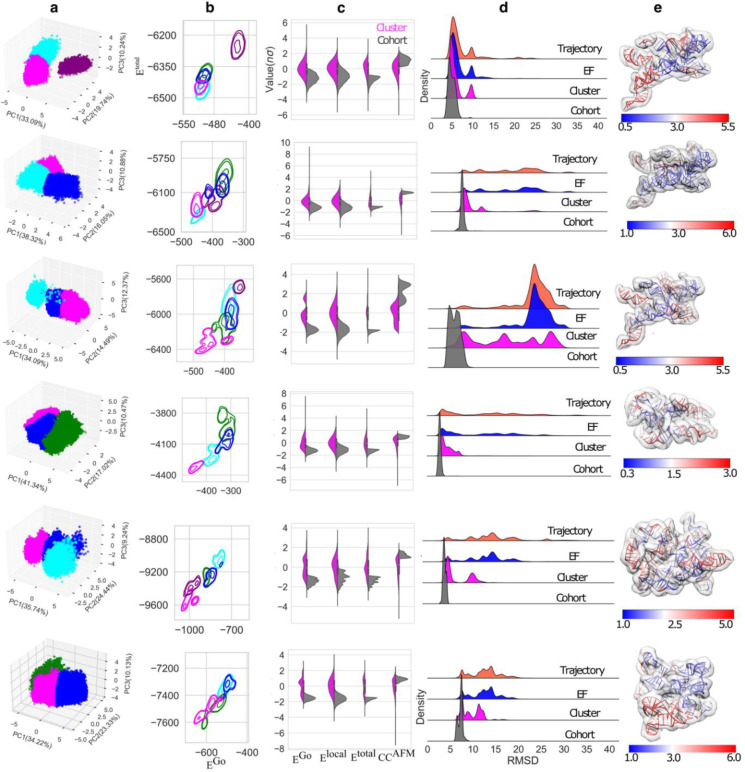
Benchmarks of unsupervised ML. **a,** 3D plot of the main representative components of PCA analysis of Benchmarks 0, 1, 2, 3, 4 and 5, respectively. For better visualization 3 populations are shown where the minimum and maximum variations are presented for each case. **b,** Contour plots for total $\left(E^{\text{Total}}\right)$ and Go $\left(E^{Go}\right)$ energies for cluster populations determined after PCA analysis, where the cluster color in magenta represents the selected representative cluster. **c,** Cohort selection applying energy and topology threshold to select models (gray) from the selected cluster (magenta). **d,** the post-unsupervised ML analysis of the progression of the selection process in terms of RMSD from the GT structures in each benchmark case, from the total trajectory population (orange), through the energy filter (blue), and clustering (magenta) to the final top cohorts (grey). The vertical axis indicates the density of populations. **e,** The top structures rendered in color in terms of RMSF compared to the GT structures.

**Fig. 4|:**
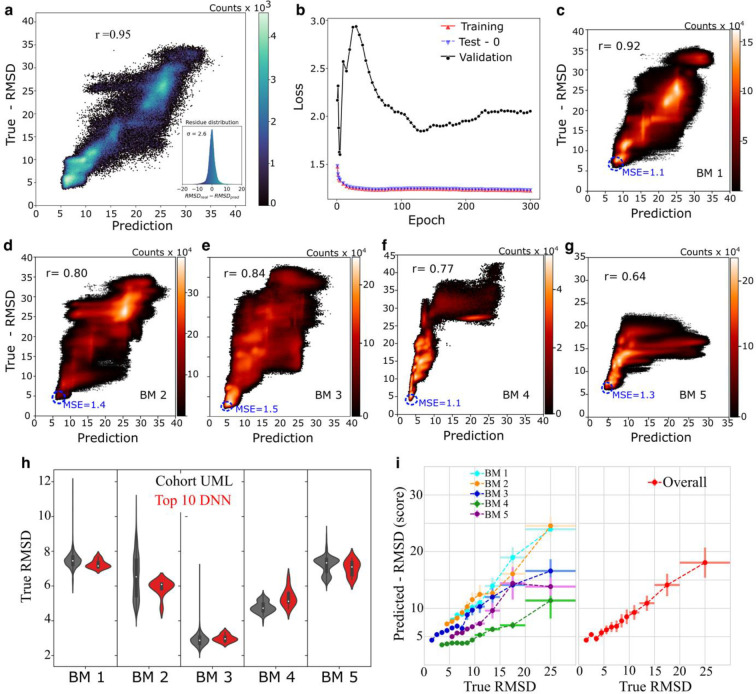
Supervised deep-learning neural network. **a,** RMSD predicted for the testing sample from the trained dataset *vs*. true RMSD with r=0.97. The inset plot shows the residues of the ${RMSD}_{\text{real}\;}-\;{\text{RMSD}}_{\text{predicted}\;}$ with σ=2.6Å. **b,** Loss as a function of the training epoch for the training set in red, validation set with RMSDs up to 10 Å in the black and initial test set in blue – the loss was evaluated after the end of each training epoch. **c-g,** The cross-validation tests using BM1–5. Numbers of structural models in each benchmark: BM1 ~ 14 million; BM2 ~ 15 million; BM3 ~ 7 million; BM4 ~ 2 million and BM5 ~ 13 million. **h**, Summary of cohort models from unsupervised ML (gray) and the top 10 models from DNN (red) in each benchmark test case. **i,** Accuracy estimation for each benchmark (left) and the average accuracy of prediction by the DNN in terms of RMSD (3–25 Å).

**Fig. 5|:**
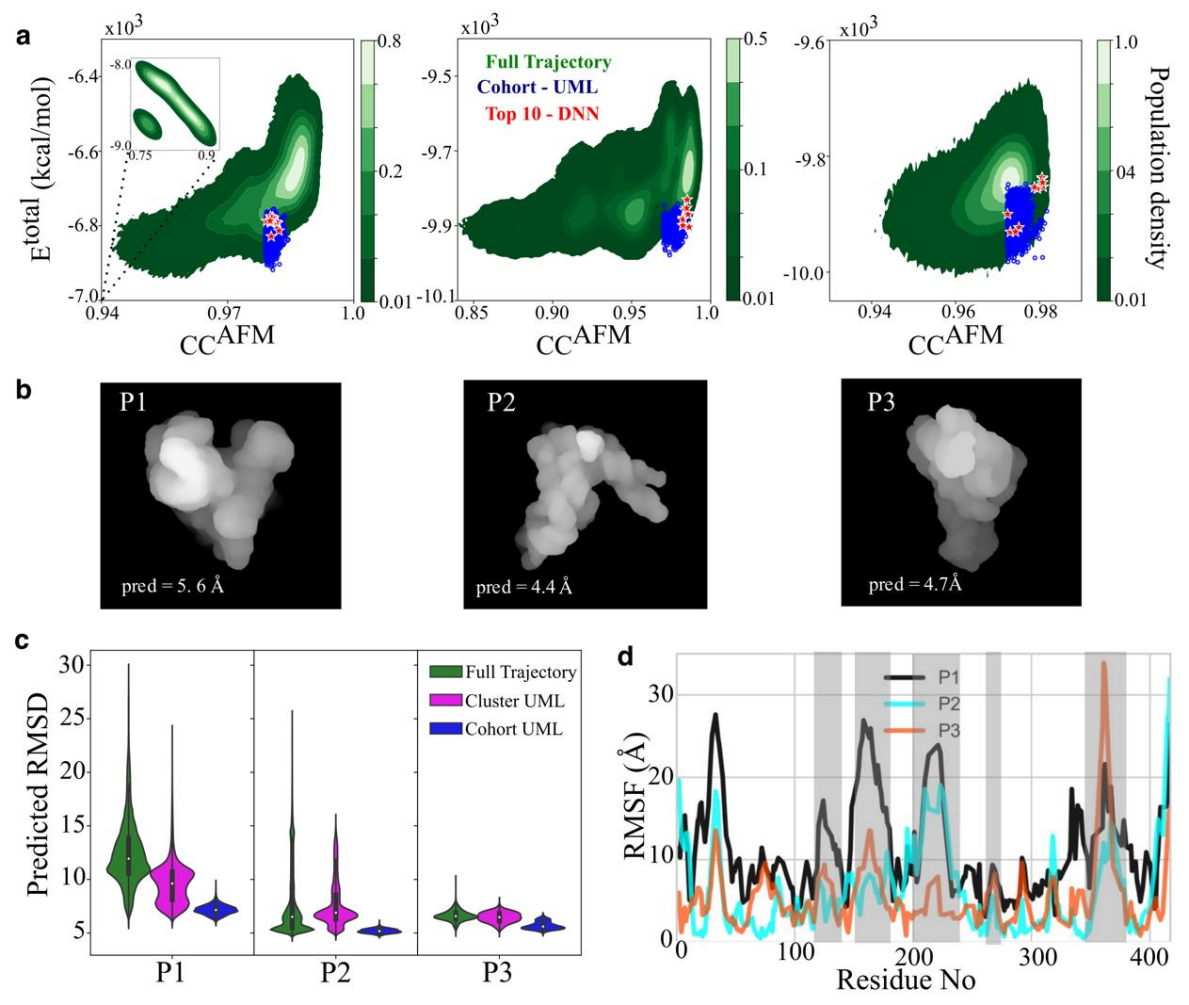
Recapitulating three topological structures from three experimental AFM particle images. **a,** Contour maps of the total energy and ${CC}^{AFM}$. The green color gradient indicates the population density, and the symbols indicate the cohort models selected by unsupervised ML (blue) and the top 10 models scored by DNN (red) for P1 (left, total 4 million models), P2 (middle, total 4 million models) and P3((right, total 2 million models). **b,** The top structural selected models rendered in molecular surface mode. **c,** DNN RMSD prediction for the full trajectory (green), and evaluation for the selected cluster and cohort from unsupervised ML for P1, P2 and P3, respectively. **d,** RMSF profiles between the crystal model and the recapitulated models of P1 (black), P2 (cyan) and P3 (orange). Residues with missing electron density in the crystal structure are highlighted in grey.

We applied our method to the adenosylcobalamin riboswitch (Cbl) crystal model (accession code: 4gma), which has a folding and size (210-nt) different from the RNA used in training and Benchmarks 1 and 2. The structure calculation was performed with a total of 20×10^6^ steps ($\left.\sim 0.9\mu s\right)$ using an AFM-topography generated with 5 Å per pixel (Extended Data Fig. 11). The final trajectory generates ~6.6 million models that were analyzed using UML and DNN (Figs. 3 and 4).

#### Benchmark 4 and Benchmark 5

These two benchmarks were designed to test our method using the initial models determined by low-resolution experimental data. In this case, we used the topological structures of RNase P RNA (298-nt) and group II intron (387-nt) ^43^ determined by using the secondary structure and small-angle X-ray scattering (SAXS) data ^37^. The GT AFM images were calculated using the crystal models for the RNAs with a resolution of 5 Å per pixel, pdb id 2A64 ^36^ and 4E8K ^35^, respectively (Extended Data Fig. 12). The structure determination was performed using a trajectory with a total of 60×10^6^ steps ($\left.\sim 2.7\mu s\right)$. The final trajectory generates ~13.4 million models that were analyzed using UML and DNN (Fig. 3 and 4).

## Data Availability

all software and scripts are available at https://github.com/PNAI-CSB-NCI-NIH/HORNET; all data for the calculations are available at https://home.ccr.cancer.gov/csb/pnai/data/HorNet/
